# Locus of Control and Self-Esteem as Predictors of Maternal and Child Healthcare Services Utilization in Nigeria

**DOI:** 10.3389/frhs.2022.847721

**Published:** 2022-04-28

**Authors:** Josephine Aikpitanyi, Friday Okonofua, Lorretta Ntoimo, Sandy Tubeuf

**Affiliations:** ^1^Institute of Health Research and Society (IRSS), Université catholique de Louvain, Ottignies-Louvain-la-Neuve, Belgium; ^2^Centre of Excellence in Reproductive Health Innovation, University of Benin, Benin, Nigeria; ^3^Department of Demography and Statistics, Federal University Oye Ekiti, Ekiti, Nigeria; ^4^Institute of Economic and Social Research (IRES), Université catholique de Louvain, Ottignies-Louvain-la-Neuve, Belgium

**Keywords:** behavioral economics, non-cognitive traits and healthcare utilization, locus of control and self-esteem, health-seeking behavior, maternal and child healthcare

## Abstract

This study investigated the influence of locus of control and self-esteem on the utilization of maternal and child healthcare services in Nigeria. Specifically, it explored the differences in utilization of antenatal care, skilled birth care, postnatal care, and child vaccination by women having internal and external locus of control and women having high and low self-esteem. It also examined the association between utilization of maternal and child healthcare on other sociodemographic characteristics. We collected information on non-cognitive traits of 1,411 randomly selected women along with information on utilization of various indicators of maternal and child healthcare services. We estimated logistic regression models for various components of maternal and child healthcare services utilization and found that women's internal locus of control was a significant predictor of utilization of antenatal care, skilled birth care and completion of child vaccination. We also found that having a high self-esteem was a significant predictor of utilization of antenatal care, postnatal care and completion of child vaccination after adjusting for other control variables. By improving our understanding of non-cognitive traits as possible barriers to maternal and child healthcare utilization, our findings offer important insights for enhancing participants' engagement in intervention programs that are initiated to improve maternal and child health outcomes in lower-middle-income countries.

## Introduction

While motherhood is expected to be a positive and joyful experience, for too many women it is correlated with suffering, disabilities and even death. Every day, 800 women die from conditions associated with pregnancy and childbirth resulting in an estimated 300,000 maternal deaths worldwide per year (1). Over 99 percent of all cases of maternal deaths occur in lower-middle-income countries, with more than half of them occurring in Sub-Saharan Africa (2). Women of childbearing age in Sub-Saharan Africa are challenged by significant medical and socio-demographic factors that translate to poor health outcomes including high maternal morbidity and deaths (3). Nigeria is the most populous nation in Africa and as a result, bears a high burden of worsening maternal and child health outcomes with a maternal mortality rate of 917 per 100,000 live births and child mortality rate of 117 per 1,000 live births (4). Major indicators of maternal and child healthcare services utilization such as antenatal care attendance, skilled assistance during childbirth, postnatal check, and child vaccination show disparities across the country. These within-country disparities cut across states, geographical regions and socioeconomic status with current statistics indicating that 77% of women in rural areas do not utilize skilled birth care during childbirth when compared with 37% of women in urban areas (5).

Findings from empirical research in several lower-middle-income countries show that avoiding maternal deaths and complications is possible when women have unhindered access to antenatal care, skilled assistance during childbirth, and postnatal care after delivery (5). Findings also show that a myriad of childhood diseases and deaths could be avoided by completing the required child vaccinations (6). Several factors have been identified as barriers to utilization of maternal and child healthcare services in lower-middle-income countries with a large proportion of studies citing lack of finances as obstacles (7, 8). This is evident in the lack of adequate healthcare financing mechanisms, resulting in high out-of-pocket costs of care. Other barriers include lack of transportation to healthcare facilities, low quality of care, lack of trust in healthcare providers, and non-availability of healthcare facilities (9).

There have been various programmes initiated to improve maternal and child health outcomes in Nigeria. These comprise components from both supply and demand of healthcare. The supply-side is focused on expanding access to quality maternal healthcare services and improving maternal and child health outcomes through the deployment and training of skilled midwives and community health workers, increasing supplies and medicines to healthcare centers, improving infrastructural development, and supporting the creation of ward development committees in rural communities (10). The demand-side is focused on increasing the utilization of healthcare services during pregnancy and childbirth by providing conditional cash transfers to pregnant women at public primary healthcare facilities (11). While these initiatives have recorded significant successes, there have been instances where the desired effects were not achieved as studies have reported that a share of women who received intervention packages, including conditional cash transfers in low resource settings continued not utilizing skilled maternity care. For instance, Baba-Ari et al. in a study to identify factors that influenced the uptake of maternal healthcare interventions found that provisions of cash transfers were not sufficient to increase uptake of maternal healthcare services in Northern Nigeria (12). McConnell et al. also found that interventions to reduce financial barriers were not enough to encourage uptake of skilled birth care in Kenya (13). Similarly, Cohen et al. found that even with the provision of cash transfers, many women in Kenya continued using poor-quality healthcare facilities for childbirth (14).

Several studies have sought to explain this phenomenon. For example, Stoop et al. reported that low levels of trust in local and national authorities were important contributors to vaccine hesitancy and hindered the uptake of child vaccinations in lower-middle-income countries (15). Smith et al. in a study to investigate the causes and covariates of antenatal care access identified late recognition of pregnancy as one of the major reasons for delayed antenatal care attendance among pregnant women in South Africa (16). Other studies have cited lack of decision-making power on the part of women, perceived low quality of care, women's preference for cultural, traditional, or religious forms of care, and fear of cesarean sections as reasons for non-utilization of maternal healthcare services in low resource settings (17, 18). Extending these past studies, we postulate that the non-utilization of maternal and child healthcare services in low resource settings could be related to intrinsic behavioral and non-cognitive traits that hinder women's ability to make rational decisions to improve their health and welfare.

While the study of cognitive traits on health-promoting behaviors has assumed increasing importance over the past years, very little is known about the influence of non-cognitive traits on health-promoting behaviors of reproductive age women. Empirical research from psychology and economics shows that non-cognitive traits are important predictors of individuals' economic and social outcomes (19). Experts are of the opinion that while the role of cognition in information processing, learning, and decision-making is essential, other non-cognitive traits also matter for attaining better life outcomes (19). The origin of the study of non-cognitive traits lies in the earlier works of sociologists Samuel Bowles and Herbert Gintis (20) in their research on the determinants of education in America. They used the phrase to distinguish factors other than those measured by cognitive tests such as the ability to read, write and apply simple numerical concepts (21). These included a wide range of traits such as empathy, resilience, locus of control, self-esteem, and the Big Five personality traits (21).

In this paper, we focused on locus of control and self-esteem because of their reference in empirical studies as important predictors of health outcomes. Economists have studied the effects of locus of control and self-esteem on socioeconomic and health-related outcomes such as dieting and engaging in physical exercises and have reported significant associations (22, 23). Locus of control is described as one of the most researched psychological concepts that exert a great impact on the decision-making ability of an individual. This could be reflected in the utilization of healthcare services or in any other field of life. Developed by the psychologist Julian Rotter in his social learning theory of personality, locus of control examines how a person perceives the relationship between his or her behavior and the expected reward (24). People who believe that they are in control of their destinies have an internal locus of control and are referred to as “internals” while people with external locus of control believe that their fates are determined by luck, chance, or powerful others and are referred to as “externals” (24).

Self-esteem involves self-evaluation followed by an emotional reaction toward oneself. Researchers describe self-esteem as a combination of a person's self-assessment and their self-concept of characteristics and abilities (25). In the last two decades, researchers have studied self-esteem in relation to problems such as drug abuse, unemployment, crime, and violence. Recent studies, have related the concept to other aspects, such as health and well-being, academic success, and learning abilities (26). In psychology, self-esteem has gained increased attention, because several studies indicate its role in important life outcomes, such as physical health, interpersonal relationships, and psychopathology (27).

Given the high rate of maternal and child morbidity and mortality in lower-middle-income countries and the large disparities in the utilization of healthcare services, examining the influence of locus of control and self-esteem on utilization of maternal and child healthcare services becomes relevant. With renewed interest in improving health outcomes exacerbated by the emergence of more infectious diseases, empirical evidence accumulates regarding the impact of psychological behaviors on health and the growing need to understand the predictors of health-promoting behaviors in low resource settings. More knowledge is needed to understand the relationship between non-cognitive variables such as locus of control and self-esteem in the utilization of maternal and child healthcare services among reproductive age women in low resource settings. The objective of this paper is to contribute to the emerging field of behavioral research and empirically evaluate whether and how locus of control and self-esteem act as predictors of the utilization of maternal and child healthcare services in Nigeria.

The rest of the paper proceeds as follows. Section 2 provides a summary of previous literature on locus of control and self-esteem. Section 3 describes our study design, empirical strategy, and key features of the chosen outcome, explanatory and control variables, along with their measurements. Section 4 presents our results. In the following section, we utilize regression models to find possible associations between locus of control and self-esteem and utilization of antenatal care, skilled birth care, postnatal care, and completion of child vaccination and we comment on the associated results. Section 5 discusses findings from the study and concludes with some final remarks.

## Locus of Control, Self-Esteem and Health-Related Outcomes

### Internal and External Locus of Control

Locus of control is described as a psychological concept that captures “*a generalized attitude, belief or expectancy regarding the nature of the causal relationship between one*'*s own behaviour and its consequences*” (24). Individuals hold beliefs on whether life outcomes are a result of their efforts or the result of fate, chance, luck, or the intervention of others that they regard as more powerful than they are. Individuals who believe that outcomes are due to their efforts have an “internal” locus of control while individuals who believe that outcomes are due to luck have an “external” locus of control (24). Locus of control is perceived as a stable trait and is described as one of the most enduring ideas in behavioral research and theory (28). People who have external locus of control attribute life outcomes to external forces such as chance, luck, fate, destiny, or powerful others, while those with internal locus of control take responsibility for their successes and failures (24).

Rotter hypothesized that individuals develop a sense of control when they perceive reinforcement as based on their behavior, with behaviors that result in reinforcement serving to strengthen their perception of control (24). However, when there is a failure of reinforcement, a reverse effect occurs that weakens or diminishes an individual's perception of control. Carton and Nowicki reviewed the research on the antecedents of individual differences in locus of control and reported that parents influenced the development of their children's locus of control (29). They found that consistent use of reward and punishment as well as encouragement of autonomy by parents were associated with the development of internal locus of control. They also found that children who had less supportive parents and those who had experienced stressful and disruptive life events had external locus of control (29). Specifically, they reported that individuals who were internally oriented in their locus of control tended to exhibit greater productivity, motivation and initiative, and were more successful in life (29).

Empirical studies have described locus of control as an important predictor of different life outcomes, including healthy living and wellbeing, life satisfaction, educational attainment, employment and wages, and could act as a buffer against many negative life events that people may experience (23, 28, 29). For example, Heckman et al. found that locus of control played a significant role in explaining risky behaviors of adolescents and young adults, including imbibing habits such as daily smoking, use of drugs, crime participation, and incarceration (30). Lassi et al. in their study on the association between locus of control and tobacco and alcohol consumption among young adults in South West England also found that individuals with more external locus of control had higher odds of consuming more tobacco and alcohol when compared with individuals with more internal locus of control (31).

Cobb-Clark et al. in their study to find the connection between individuals' healthy habits and their locus of control in Australia reported that people with internal locus of control tended to invest more in their health by imbibing healthier lifestyles (22). Gale et al. also found that individuals with internal locus of control had reduced risk of obesity and reported higher satisfaction with their health (32). Kesavayuth et al. in their study to find the impact of locus of control on healthcare utilization in Australia also found that people with internal locus of control were healthier than those with external locus of control were and relied less on both preventive and curative medical care (23).

### High and Low Self-Esteem

William James first covered the concept of self-esteem in 1890 (33). He defined it as “*a positive self-consideration obtained by people when they can consistently meet or exceed the important goals of their lives*” (33). Morris Rosenberg, the author of one of the oldest and most widely used self-esteem evaluation scales defined self-esteem as, “*a positive or negative overall attitude towards oneself* ” (34). Murphy et al. related self-esteem to individuals' personal beliefs about social relationships, skills and abilities; they defined self-esteem as “*a global barometer of self-evaluation involving cognitive appraisals about general self-worth and affective experiences of the self that are linked to these global appraisals*” (35). Scholars have adopted a two-way approach in the evaluation of self-esteem. This two-way approach divides self-esteem into the categories of high self-esteem, defined as “*when a person feels that they have value for self* ” and low self-esteem, characterized as “*when a person believes that they have no self-value and, as a result, suffer from self-pity and self-contempt*” (36).

An important consequence of the self-esteem situational variability is that individuals with different levels of self-esteem tend to apply opposite behavioral strategies in front of at-risk situations. While individuals with high self-esteem exhibit self-enhancing tendencies, individuals with low self-esteem tend to use self-protective strategies to avoid the attention of other people, and to conceal their inadequacies. Moreover, low self-esteem individuals are highly sensitive to defeats and interpersonal refusals, as they are often less able to use their mental resources as a defensive instrument to avoid dramatic fluctuations (37). Empirical and theoretical studies over the last two decades have identified self-esteem as a powerful and significant psychological factor in individuals' overall quality of life, and in determining healthy living and well-being.

Studies have found that feeling worthy and empowered are related to having a high self-esteem, which can result in positive lifestyle changes and improve the tendency to have better health outcomes (38). For example, Das and Pattanaik found that individuals with high self-esteem saw themselves as being more active and capable to influence their lives through personal efforts and the setting of higher life goals (39). Similarly, Fuscaldi et al. reported that diabetic patients with higher self-esteem easily adopted healthier behaviors concerning the diagnosis and treatment of the disease when compared with patients with lower self-esteem (40). Baumeister et al. also identified low self-esteem as a risk factor for aggression, depression, felony and lower educational outcomes (26). These findings showed that individuals who regarded themselves highly and had high self-esteem were more likely to be involved in health-promoting behaviors, and that individuals who regarded themselves lowly and had low self-esteem were less likely to engage in health-promoting behaviors.

### Locus of Control and Self-Esteem

Several researches and theories suggest the existence of a strong relationship between locus of control and self-esteem. Empirical research on the association between locus of control and self-esteem have identified that locus of control was significantly related to self-esteem on the dimension of individuals' control ideology, self-blame and system blame, suggesting that having an internal locus of control was associated with having high self-esteem (37, 38). For instance, Giblin et al. found that individuals that had internal locus of control also had high self-esteem (41). Dielman et al. reported a similar relationship between attribution for outcome and self-esteem. They reported that individuals with high self-esteem attributed successful outcomes to internal causes, while individuals with low self-esteem attributed successful outcomes to external causes (42). Alizadeh also found positive significant correlations between internal locus of control and self-esteem. They found that individuals with internal locus of control also had high self-esteem and had better physical and mental health, while individuals with external locus of control also had low self-esteem and were psychologically distressed and perhaps even depressed (43).

Recognizing the potential influence of locus of control and self-esteem on health and well-being raises important questions about the variability of both measures in any given population. From a theoretical angle, researchers have found that both traits were shaped within the context of the social environment and were likely to be formed by an individual's position in the social structure (38, 39). While several of these past studies have been based on finding the influence between locus of control and self-esteem on health outcomes and other aspects of healthcare, we did not find any study on the influence of these traits on utilization of skilled maternal and child healthcare services. Our study becomes very relevant in being one of the first to find the relationship between these traits and utilization of maternal and child healthcare services with recommendations for designing effective interventions, especially in low resource settings.

## Methods

### Study Design and Population

We used data from an intervention project aiming to increase women's access to skilled maternal healthcare services that was conducted in two rural Local Government Areas (Esan South East and Etsako East) in Edo State, southern Nigeria between August 2017 and June 2020. The two Local Government Areas have a total population of 455,432 persons, with Esan South East accounting for 241,492 and Etsako East LGA accounting for 213,940 (44). Both LGAs are located in the rural and riverine areas of the state, adjacent to River Niger, with Estako East in the northern part of the Edo State part of the river, while Esan South East is in the southern part. Administratively, each LGA comprises of 10 political/health wards and there are several communities in each ward. Nigeria operates a three-tier health care system with primary healthcare as the entry into the health system (45). The principal sources of maternal and child healthcare in the two local government areas are primary healthcare centers. The sampling technique used to select communities and respondents in each local government area is presented in detail in a previous publication (46). A sample of 20 communities was targeted and a sample of 1411 women of reproductive age (15 – 45 years) that gave birth in two years prior to the survey were selected to participate in the project. Trained research assistants administered questionnaires through face-to-face interviewing of respondents.^1^ The questions were fielded in English or Pidgin English as appropriate.

The intervention consisted in providing financial assistance to pregnant women in form of a community-based health insurance scheme that subsidized the cost of delivery by 80%. Additionally, women in the programme were provided with mobile phones with dedicated SIM cards linked to health care providers and transporters to ease access to health care facilities. Advocacy visits were made to relevant authorities to ensure that health care providers (doctors and nurses) were posted to the various health care centers and arrangements were made with local transporters in collaboration with the health care providers to transport women to health care facilities freely and when they called. Although the intervention activities were available to all women of reproductive age within the community, financial assistance for childbirth and mobile phones with dedicated SIM cards were given to only women that were registered in the community-based health insurance scheme.

We utilized two data collection points. The baseline questionnaire carried out before the intervention in 2017 consisted of pre-validated questions adapted from the Nigeria Demographic and Health Survey (47). It included questions on women's socio-demographic characteristics, partners' and other family characteristics, reproductive history, and antenatal, intrapartum, and postnatal care experience for current pregnancy and births in the preceding years. The baseline questioned women on reasons for use, non-use of primary health centers for maternal, and child healthcare services and identified barriers to utilization of maternal healthcare services from a list of factors. These included financial constraints, lack of transportation to healthcare facilities, inadequate number of trained health care providers, and lack of drugs and other needed consumables at healthcare facilities (48). In addition to the repeated baseline survey questionnaire conducted at follow-up in July 2020, we added a set of standardized questions on locus of control and self-esteem to investigate whether and how women's non-cognitive traits mattered for the utilization of maternal and child healthcare services. In this paper, we utilized data from the follow-up survey, which contained information on women's locus of control and self-esteem.

### Empirical Strategy

To understand the theoretical aspects of individuals' healthcare-seeking behavior, we looked at the Grossman model of demand for health (49). This model was constructed within a human capital framework and categorizes health as a durable capital stock that depreciates over an individual's life cycle and could be increased by investing in health inputs such as consuming medical services and imbibing a healthy lifestyle (exercise, recreation, diet). Grossman opined that individuals demanded “good health” for two reasons. First, to increase the number of healthy days that allowed for market and non-market activities (here, good health is considered as an investment good). Second, to improve welfare or utility (here, good health is seen as a consumption good).

In the Grossman model, demand for healthcare services is derived from the demand for health. This implies that factors, which influence demand for good health also influence demand for medical services and healthy lifestyles. Thus, individuals do not demand medical services for their own sake, but as a means to attain good health (49). Individuals consume healthcare services in order to improve their health status, so the cost of gaining good health is a reduction in the consumption of other goods and they could choose the type of healthcare to consume at any point in time using available information, by weighing the costs of utilization to the perceived benefits of utilizing healthcare services. In our study, we proposed that a pregnant mother might consume healthcare services, not just to improve her own health but also to improve that of her unborn child. This could be achieved through early registration for antenatal care and attending at least four antenatal care visits as recommended by the World Health Organization (50), utilizing the services of a skilled birth attendant during childbirth, receiving postnatal care within 48 h of childbirth and completing the required child vaccinations. Thus, not consuming these healthcare services might result in worsened health outcomes for both mother and child.

In the Grossman model, the role of cognitive skills is specified through its analysis of the effect of education on health outcomes. In this study, we argued that the claims made about education levels were applicable to non-cognitive traits as well. This is in line with the findings of Chiteji in a study conducted in the USA on the influence of non-cognitive skills in encouraging healthy behavior. The study suggested that non-cognitive traits might raise the efficiency of household production just as education does in the standard Grossman model (51). We based our assumptions that in a country setting such as that of Nigeria, where women occupy marginal positions in society, their healthcare-seeking behavior might be affected by a myriad of psychological, physical and behavioral factors. Thus, we analyzed the demand-side factors, which are expected to influence healthcare service utilization, and provide a general perspective based on which, we might formulate hypotheses regarding which variables affect maternal and child healthcare-seeking behavior. In this study, our intent was to establish the influence of locus of control and self-esteem as demand-side predictors of maternal and child healthcare services utilization. The outcome variables, *y*_*i*_, thus took a binary form, where;

*y*_*i*_ = 1, if individual *i* utilized healthcare
*y*_*i*_ = 0, if individual *i* did not utilize healthcare

When dealing with binary responses, it is not appropriate to use ordinary least squares models because the standard assumptions are not satisfied. Instead, non-linear binary choice models such as logit and probit models are more suitable for analysis (52). Logit and probit models give very similar results in empirical work, but since most previous research of healthcare utilization uses the logit model, we followed the same line in this study. The probability that a given individual utilized healthcare services, i.e., that the outcome variable took the value 1, given a set of explanatory variables, could be written as


(1) $$\begin{array}{r} P\left(y_{i}=1/X_{i}\right) \end{array}$$

This probability could be transformed into;


(2) $$\begin{array}{r} \frac{P\;\left(yi=\frac{1}{Xi}\right)}{P\;\left(yi=\frac{0}{Xi}\right)}\;=\;\frac{P\;\left(yi=\frac{1}{Xi}\right)}{1-\;P\;\left(yi=\frac{1}{Xi}\right)} \end{array}$$

which indicated how often an event (*y*_*i*_ = 1; the individual utilized health care) occurred, relative to how often it did not occur (*y*_*i*_ = 0; the individual did not utilize health care).

Following the theoretical framework, we employed logit regression models to assess the association between non-cognitive traits and utilization of maternal and child healthcare. For each of the four indicators of healthcare utilization *y*_*i*_, we estimated models of the form:


(3) $$\begin{array}{r} {\text{y}}_{\text{i}}=\alpha +\beta {\text{T}}_{\text{i}}+\gamma {\text{X}}_{\text{i}}+\varepsilon_{\text{i}} \end{array}$$

Where *i* indicated the individual, T indicated traits (locus of control and self-esteem) and X indicated all other control variables, as described in Table 1. We presented results as beta coefficients, with standard errors. With four outcomes and two non-cognitive traits, we adjusted our results for multivariate regressions to enable us identify as many significant associations as possible.

**Table 1:** Measurement of variables.

**Variables**	**Descriptions**
**Outcome variables**	
Antenatal care	This was derived from response to the question “Did you see anyone for antenatal care when you were pregnant with your last child?” It was computed as “0” = 'No' and “1” = “Yes”.
Skilled birth care	This was derived from response to the question “Who assisted with the delivery of your last child?” It was computed as “0” = No skilled birth attendant (doctor, trained nurse or midwife) during last childbirth and “1” = Skilled birth attendant during last childbirth.
Postnatal check	This was derived from the question “Did anyone check on your health within 48 h after the delivery of your last child?” It was computed as “0” = “No” and 1 = “Yes”.
Child vaccination	This was derived from the question “Did you complete the vaccination for your last child?” It was computed as “0” = “No” and “1” = “Yes”.
**Explanatory variables**	
Locus of control	Locus of control was derived from the responses to seven questions. Answers were reported on a 5-point scale that ranged from 1 = strongly disagree to 5 = strongly agree
Self-esteem	Self-esteem was derived from the responses to 10 questions. Answers were reported on a 5-point scale that ranged from 1 = strongly disagree to 5 = strongly agree
***Sociodemographic characteristics***	
Age	Grouped in categories of 15–24, 25–34, 35–44 and >44
Education	Grouped as no education, primary education, secondary education and higher education
Marital status	Grouped as married and not married
Occupation	Grouped as working and not working
Literacy	Grouped as cannot read, can read a bit and can read well
Religion	Grouped as Christianity, Islam and Others
***Household characteristics***	
Family type	Grouped as monogamy and polygamy
Partner's age	Grouped in categories of 18–37, 38–57, 58–77 and >77
Partner's education	Grouped as no education, primary education, secondary education and higher education
Partner's occupation	Grouped as working and not working
Decision on healthcare	Grouped as Respondent herself, Partner, Both and Others
Payment for healthcare	Grouped as Respondent herself, Partner, Both and Others
Previous pregnancy complications	Yes = If respondent had complications in previous pregnancies and No = If respondent had no previous pregnancy complications

We followed Kesavayuth et al. and Buddelmeyer and Powdthavee that recommended regressing non-cognitive traits on sociodemographic variables. We regressed non-cognitive traits *T* (locus of control and self-esteem) on women's sociodemographic variables that were included as control variables in the model in order to determine the sociodemographic factors that influenced locus of control and self-esteem using a linear regression model (23, 53).


(4) $$\begin{array}{r} T=\beta_{0}+\beta_{1}X_{1}+e \end{array}$$

Where *X*_1_ was a vector of women's sociodemographic variables and e the random error term.

### Outcome Variables

#### Measures of Healthcare Services Utilization

Our outcome variables included various indicators of maternal and child healthcare utilization. In total, we examined four indicators (antenatal care, skilled birth care, postnatal care, and completion of child vaccination). Our measure of antenatal care differed from the World Health Organisation's recommendation of having “at least four antenatal care visits” (50). This was because a large number of women in our study population were unable to provide information on the number of times that they received antenatal checks. As a result, we collected data on antenatal care as a binary variable in response to the general question “Did you see anyone for antenatal care during your last pregnancy?” the variable was coded as “0” if the response was “No” and “1” if the response was “Yes”.

Utilization of skilled birth care was derived as a response to the question “Who assisted with the delivery of your last child?” It was coded as “0” if no skilled birth attendant (doctor, trained nurse or midwife) was present and “1” if a skilled birth attendant was present. Utilization of postnatal care was derived from the question “Did anyone check on your health within 48 hours after the delivery of your last child?” It was computed as “0” if the response was “No” and “1” if the response was “Yes”. Completion of child vaccination was derived from the question “Did you complete the vaccination of your last child?” It was computed as “0” if the response was “No” and “1” if the response was “Yes”. Table 1 gives a summary description of all outcome, explanatory, and control variables.

### Explanatory Variables

#### Measures of Non-cognitive Traits

The explanatory variables of interest were “locus of control” and “self-esteem”. We asked participants all seven of the original items from the Psychological Coping Resources component of the Mastery Module developed by Pearlin and Schooler. Mastery refers to beliefs about the extent to which life's outcomes are under one's own control using the following statements “*(a) I have little control over the things that happen to me*; *(b) There is really no way I can solve some of the problems I have*; *(c) There is little I can do to change many of the important things in my life*; *(d) I often feel helpless in dealing with the problems of life*; *(e) Sometimes, I feel that I am being pushed around in life*; *(f) What happens to me in the future mostly depends on me*; *(g) I can do just about anything I really set my mind to do*;” along with answers on a Likert scale from 1 to 5 (54).

Specifically, respondents were asked the extent to which they agree with the seven statements. Possible responses ranged from 1 (*strongly disagree*) to 5 (*strongly agree*). In line with Cobb-Clark et al. and Kesavayuth et al., which recommended generating an index variable for locus of control, we generated an index variable for locus of control using a factor analysis method. Factor analysis is a statistical technique that linearly transforms an original set of variables into a substantially smaller set of uncorrelated variables that represent most of the information in the original set of variables. An internal test of consistency yields a Cronbach's reliability statistic of 0.78 indicating that the seven items were highly reliable (55). We generated a single index for the locus of control variable by reversing the scores of the responses to questions 1 through 5, and then adding the scores of questions 6 and 7. The total score thus ranged from 5 to 35 with higher values indicating a more internal locus of control.

We measured self-esteem with the scale developed by Morris Rosenberg in 1965 (33). It consists of 10 items including the following statements “*(a) I feel that I am a person of worth; (b) I feel that I have a number of good qualities; (c) I am inclined to feel that I am a failure; (d) I am able to do things as well as most people; (e) I feel that I do not have much to be proud of; (f) I take a positive attitude toward myself; (g) On the whole, I am satisfied with myself; (h) I certainly feel useless at times; (i) I wish I could have more respect for myself; (j) At times, I think that I am no good at all*.” Respondents were asked the extent to which they agreed with the ten statements with possible answers on a 5-point scale from 1 (*strongly disagree*) to 5 (*strongly agree*). Five statements were positively scored whereas the rest five statements were negatively scored. Following Kaplan and Pokorny, and Das and Pattanaik we generated an index score for self-esteem using a factor analysis method (39, 56). We reversed the negative scores and added them to the positive scores to generate a self-esteem index with higher scores indicating higher self-esteem. A Cronbach alpha score of 0.70 indicates reliability of the scale. The Rosenberg self-esteem scale is considered reliable and a valid tool for quantitative self-esteem assessment (26, 35, 37).

### Control Variables

In analyzing the influence of non-cognitive traits on maternal and child healthcare utilization, it is important to control for other differences in observable characteristics that may influence the use of healthcare services. The Anderson framework, which highlights the importance of predisposing, enabling and health need variables in explaining healthcare utilization (57), was useful for this purpose. We therefore adopted the Anderson framework in choosing appropriate control variables; predisposing factors include indicators for women's age, education level, marital status, level of literacy, religion and parity. Enabling characteristics included household characteristics such as whether a woman was in a monogamous or polygamous union, partners' age, partners' education level, partners' occupation, ability to make decisions on healthcare use and ability to pay for healthcare. The indicator of “health need” included having had a previous pregnancy complication. We checked for correlations between indicators before including them in the models.

### Data Analysis

We coded the obtained data and entered them into Stata 17.0 for Windows. We calculated means and standard deviations for continuous variables (locus of control and self-esteem). We also calculated frequencies and percentages for categorical variables. We performed standard descriptive analyses on the main outcome and explanatory variables. Next, we explored the correlations between the locus of control, self-esteem, antenatal care, skilled birth care, postnatal checks, and child vaccination and examined whether multicollinearity was an issue using variance inflation factors. Computed factors ranged between 1.05 and 2.20, which was sufficiently low to assume that it would not significantly affect coefficient estimates. We examined the association between non-cognitive traits (locus of control and self-esteem) and the four outcomes of the utilization of maternal and child healthcare using Chi-square tests. To begin our analysis, first, we estimated two linear regression models to determine the sociodemographic characteristics of women that were associated with locus of control and self-esteem. We then fitted four logit regression models to examine the associations between locus of control and self-esteem and the measures of maternal and child healthcare services utilization.

## Results

In Table 2, we presented the descriptive statistics (number of observations, proportion and range of sum score). Our findings revealed that 13.7% of women in the study sample did not see a skilled healthcare provider for antenatal care visits, 11.9% did not utilize skilled birth care, 7.4 % did not have postnatal check after childbirth and 5.0 % did not complete vaccinations for their children. However, utilization of various indicators of skilled maternity and child healthcare were higher in the study population than that of the national average.^2^ The correlation matrices for all these measures of maternal and child healthcare utilization, locus of control, and self-esteem are shown in Table 3. We noted a positive but medium correlation between locus of control and self-esteem (0.23). Correlations between locus of control and the measures of maternal and child healthcare utilization were negative and tended to be low (0.05–0.17). Similar negative and low correlations were found between self-esteem and the measures of maternal and child healthcare services utilization (0.08–0.02). We found medium to high correlations within the measures of maternal and child healthcare services utilization (0.34–0.85). The highest correlation was between utilization of antenatal care and skilled birth care services (0.85).

**Table 2:** Descriptive statistics of outcome and explanatory variables.

**Variables**	**Observations**	**Proportion**	**Min**	**Max**
**Outcome variables**				
Antenatal care	1,351		0	1
No		0.137		
Yes		0.863		
Skilled birth care	1,359		0	1
No		0.119		
Yes		0.880		
Postnatal care	1,358		0	1
No		0.074		
Yes		0.950		
Child vaccination	1,358		0	1
No		0.049		
Yes		0.950		
**Explanatory variables**				
Locus of control	1,411		10	35
Internal		0.111		
External		0.888		
Self-esteem	1,411		25	50
High		0.236		
Low		0.763		

**Table 3:** Spearman's Correlation matrix between locus of control, self-esteem, antenatal care, skilled birth care, postnatal care and child vaccination.

	**Locus of control**	**Self-esteem**	**Antenatal care**	**Skilled birth**	**Postnatal care**	**Child vaccination**
Locus of control	1.000					
Self-esteem	0.2334	1.000				
Antenatal	−0.1786	−0.0531	1.000			
Skilled birth	−0.1311	−0.0456	0.8503	1.000		
Postnatal	−0.0621	−0.0805	0.4136	0.4107	1.000	
Vaccination	−0.0519	−0.0277	0.3453	0.3894	0.4953	1.000

In Table 4, we presented the descriptive characteristics of women based on their locus of control and self-esteem. We found that women with external locus of control were less likely to utilize the various components of maternal and child healthcare (13.2, 11.6, 6.8, and 4.6 %) when compared to women with internal locus of control (0.6, 0.7, 0.7, and 0.4 %). We also found similar results for self-esteem. Women with low self-esteem had lower likelihoods of utilizing antenatal care, skilled birth care, postnatal care, and completing their children's vaccinations (11.0, 9.6, 6.3, and 9.6 %) when compared with women with high self-esteem (2.7, 2,6, 1.3, and 0.8 %).

**Table 4:** Description of variables based on locus of control and self-esteem.

**Variables**	**Locus of control**			**Self-esteem**		
	**Internal**	**External**	***p*-value**	**High**	**Low**	***p*-value**
	***N*** **(%)**	***N*** **(%)**		***N*** **(%)**	***N*** **(%)**	
	**157 (11.13)**	**1,254 (88.87)**		**333 (23.60)**	**1,078 (76.40)**	
**Antenatal**			0.001			0.153
No	8 (0.59)	178 (13.18)		37 (2.74)	149 (11.03)	
Yes	144 (10.66)	1021 (75.57)		288 (21.32)	877 (64.91)	
**Skilled birth**			0.010			0.338
No	9 (0.66)	157 (11.55)		35 (2.58)	131 (9.64)	
Yes	145 (10.67)	1048 (77.12)		292 (21.49)	901 (66.30)	
**Postnatal**			0.611			0.069
No	10 (0.74)	92 (6.77)		17 (1.25)	85 (6.26)	
Yes	144 (10.60)	1112 (81.89)		310 (22.83)	946 (69.66)	
**Vaccination**			0.287			0.118
No	5 (0.37)	63 (4.64)		11 (0.81)	57 (4.20)	
Yes	149 (10.97)	1141 (84.02)		316 (23.27)	974 (71.72)	
**Age**			0.000			0.005
15–24	15 (1.06)	251 (17.79)		43 (3.05)	223 (15.80)	
25–34	52 (3.69)	524 (37.14)		134 (9.50)	442 (31.33)	
35–44	50 (3.54)	354 (25.04)		109 (7.73)	295 (20.91)	
>44	40 (2.83)	125 (8.86)		47 (3.33)	118 (8.36)	
**Education**			0.000			0.254
None	12 (0.85)	165 (11.69)		43 (3..05)	134 (9.50)	
Primary	83 (5.88)	503 (35.65)		132 (9.36)	454 (32.18)	
Secondary	42 (2.98)	544 (38.55)		137 (9.71)	449 (31.82)	
Higher	20 (1.42)	42 (2.98)		21 (1.49)	41 (2.91)	
**Marital status**			0.000			0.095
Married	60 (4.25)	708 (50.18)		168 (11.91)	600 (42.52)	
Not married	97 (6.87)	546 (38.70)		165 (11.69)	478 (33.88)	
**Occupation**			0.008			0.000
Not working	23 (1.63)	303 (21.47)		110 (7.80)	216 (15.31)	
Working	134 (9.50)	951 (67.40)		223 (15.80)	862 (61.09)	
**Literacy**			0.466			0.910
Cannot read	62 (4.39)	534 (37.85)		144 (10.21)	452 (32.03)	
Can read a bit	62 (4.39)	505 (35.79)		131 (9.28)	436 (30.90)	
Can read well	33 (2.34)	215 (15.24)		58 (4.11)	190 (13.47)	
**Religion**			0.066			0562
Christianity	155 (10.99)	1195 (84.69)		319 (22.61)	1031 (73.07)	
Islam	0 (0.00)	42 (2.98)		8 (0.57)	34 (2.41)	
Others	2 (0.14)	17 (1.20)		6 (0.43)	13 (0.92)	
**Parity**			0.483			0.013
1	117 (8.29)	962 (68.18)		254 (18.00)	825 (58.47)	
2	38 (2.69)	263 (18.64)		65 (4.61)	236 (16.73)	
3	2 (0.14)	29 (2.06)		14 (0.99)	17 (1.20)	
**Family type**			0.000			0.684
Monogamy	129 (9.14)	756 (53.58)		212 (15.02)	673 (47.70)	
Polygamy	28 (1.98)	498 (35.29)		121 (8.58)	405 (28.70)	
**Partner's age**			0.016			0.019
18–37	49 (3.47)	455 (32.25)		113 (8.01)	391 (27.71)	
38–57	94 (6.66)	718 (50.89)		188 (13.32)	624 (44.22)	
58–77	14 (0.99)	55 (3.90)		27 (1.91)	42 (2.98)	
>77	0 (0.00)	26 (1.84)		5 (0.35)	21 (1.49)	
**Partner's education**			0.000			0.015
None	20 (1.42)	194 (13.75)		62 (4.39)	152 (10.77)	
Primary	67 (4.75)	312 (22.11)		100 (7.09)	279 (19.77)	
Secondary	47 (3.33)	595 (42.17)		127 (9.00)	515 (36.50)	
Higher	23 (1.63)	153 (10.84)		44 (3.12)	132 (9.36)	
**Partner's occupation**			0.569			0.000
Not working	23 (1.63)	206 (14.60)		89 (6.31)	140 (9.92)	
Working	134 (9.50)	1048 (74.27)		244 (17.29)	938 (66.48)	
**Decision on healthcare**			0.000			0.000
Self	32 (2.27)	160 (11.34)		68 (4.82)	124 (8.79)	
Partner	44 (3.12)	694 (49.18)		123 (8.72)	615 (43.59)	
Both	81 (5.5.74)	393 (27.85)		140 (9.92)	334 (23.67)	
Others	0 (0.00)	7 (0.50)		2 (0.14)	5 (0.35)	
**Payment for healthcare**			0.000			0.000
Self	35 (2.48)	191 (13.54)		73 (5.17)	153 (10.84)	
Partner	57 (4.04)	834 (59.11)		180 (12.76)	711 (50.39)	
Both	65 (4.61)	217 (15.38)		73 (5.17)	209 (14.81)	
Others	0 (0.00)	12 (0.85)		7 (0.50)	5 (0.35)	
**Previous preg. comp**			0.000			0.322
Yes	39 (2.76)	219 (15.52)		67 (4.75)	191 (13.54)	
No	118 (8.36)	1035 (73.35)		266 (18.85)	887 (62.86)	

*N, Number of observations; %, Percentage; 157, number of respondents having internal locus of control; (11.13), percentage of respondents having internal locus of control; 1,254, number of respondents having external locus of control; (88.87), percentage of respondents having external locus of control; 333, number of respondents having high self-esteem; (23.60), percentage of respondents having high self-esteem; 1,078, number of respondents having low self-esteem; (76.40), percentage of respondents having low self-esteem*.

In Table 5, we used linear regression to analyse the sociodemographic variables that predict locus of control and self-esteem. We found that locus of control was associated with age indicating that older women were more internal in their locus of control. Furthermore, we found that women who had internal locus of control were more educated, having at least secondary education, were employed and lived with a partner. Similarly, women with high self-esteem had more years of education, were employed, and lived with a partner. In the next step, we performed the regression analyses of the associations of the outcome variables with locus of control and self-esteem as described in equation (3).

**Table 5:** Determinants of locus of control and self-esteem.

**Variables**	**Locus of control**	**Self-esteem**
	**Coeff (S.E)**	**Coeff (S.E)**
**Age**		
15–24	Ref	Ref
25–34	−0.200 (0.076)^**^	−0.105 (0.033)^***^
35–44	−0.349 (0.084)^***^	−0.174 (0.036)^***^
>44	−0.635 (0.103)^***^	−0.199 (0.044)^***^
**Education**		
None	Ref	Ref
Primary	−0.081 (0.087)	−0.015 (0.037)
Secondary	0.114 (0.102)	−0.065 (0.044)
Higher	−0.444 (0.169)^***^	−0.181 (0.072)^**^
**Marital status**		
Married	Ref	Ref
Not married	−0.243 (0.053)^***^	−0.046 (0.022)^**^
**Occupation**		
Not working	Ref	Ref
Working	0.315 (0.064)^***^	0.165 (0.027)^***^
**Literacy**		
Cannot read	Ref	Ref
Can read a bit	−0.207 (0.067)^***^	0.011 (0.029)
Can read well	−0.198 (0.096)^*^	0.040 (0.041)
**Religion**		
Christianity	Ref	Ref
Islam	0.094 (0.153)	0.015 (0.065)
Others	−0.219 (0.225)	−0.074 (0.096)
**Parity**		
1	Ref	Ref
2	−0.085 (0.066)	0.012 (0.028)
3	0.040 (0.180)	−0.192 (0.077)
**Family type**		
Monogamy	Ref	Ref
Polygamy	0.083 (0.054)	0.015 (0.023)
No of obs.	1,411	1,411
R-Squared	0.0711	0.0489
Adjusted R-Squared	0.0612	0.0387

* *p < 0.05*.

** *p < 0.01*.

*** *p < 0.001*.

*Statistical significance at α = 0.05*.

We used four logit regression models to examine the associations between locus of control and self-esteem on the utilization of antenatal care, skilled birth care, postnatal care, and child vaccination, and presented our results in Tables 6, 7. In Table 6, we found negative coefficients of locus of control for all outcome variables indicating an inverse relationship between locus of control and utilization of various components of maternal and child healthcare services. This meant that women with more external locus of control had lower likelihood of utilizing maternal and child healthcare services when compared with women with more internal locus of control. The estimated coefficients on locus of control were statistically significant in the utilization of antenatal care, skilled birth care, and child vaccination. We found the coefficient of locus of control not statistically significant in the utilization of postnatal care. In Table 7, we also found an inverse relationship between self-esteem and utilization of various components of maternal and child healthcare services as shown by the negative coefficients indicating that women with low self-esteem were less likely to utilize maternal and child healthcare services when compared with women with high self-esteem. We found this reflected in the estimated coefficients of self-esteem on utilization of postnatal care and completion of child vaccination, as they showed statistically significant coefficients. We however found the estimated coefficients of self-esteem not statistically significant on utilization of antenatal care and skilled birth care after adjusting for other control variables.

**Table 6:** Locus of control and utilization of antenatal care, skilled birth care, postnatal care and child vaccination.

**Variables**	**Antenatal care**	**Skilled birth care**	**Postnatal care**	**Child vaccination**
	**Coeff (S.E)**	**Coeff (S.E)**	**Coeff (S.E)**	**Coeff (S.E)**
Locus of control	−0.430 (0.104)^**^	−0.333 (0.104)^***^	−0.144 (0.116)	−0.305 (0.149)^**^
**Age**				
15–24	Ref	Ref	Ref	Ref
25–34	0.152 (0.271)	0.027 (0.286)	0.160 (0.344)	0.257 (0.411)
35–44	0.265 (0.319)	0.027 (0.335)	−0.439 (0.414)	−0.331 (0.503)
>44	0.491 (0.429)	0.400 (0.457)	0.015 (0.537)	0.486 (0.702)
**Education**				
None	Ref	Ref	Ref	Ref
Primary	0.400 (0.254)	0.366 (0.265)	−0.554 (0.402)	0.403 (0.432)
Secondary	0.976 (0.329)^***^	0.879 (0.342)^**^	−0.525 (0.482)	0.277 (0.537)
Higher	0.693 (0.669)	0.351 (0.645)	−0.849 (0.739)	−0.818 (0.854)
**Marital status**				
Married	Ref	Ref	Ref	Ref
Not married	0.660 (0.190)^***^	0.764 (0.201)^***^	−0.436 (0.234)	−1.025 (0.295)^***^
**Occupation**				
Not working	Ref	Ref	Ref	Ref
Working	−0.161 (0.248)	0.010 (0.252)	−0.592 (0.335)	0.084 (0.357)
**Literacy**				
Cannot read	Ref	Ref	Ref	Ref
Can read a bit	−0.031 (0.210)	−0.046 (0.221)	0.583 (0.282)^**^	0.036 (0.344)
Can read well	0.321 (0.348)	0.349 (0.369)	0.207 (0.394)	0.513 (0.538)
**Religion**				
Christianity	Ref	Ref	Ref	Ref
Islam	−0.326 (0.427)	−0.594 (0.413)	0.392 (0.761)	−0.272 (0.775)
Others	−0.446 (0.604)	0.727 (0.826)	−0.029 (0.939)	−0.769 (0.826)
**Parity**				
1	Ref	Ref	Ref	Ref
2	0.031 (0.216)	0.164 (0.231)	0.753 (0.336)^**^	0.152 (0.346)
3	−0.178 (0.583)	−0.010 (0.650)	−0.971 (0.558)	−0.409 (0.790)
**Family type**				
Monogamy	Ref	Ref	Ref	Ref
Polygamy	−0.232 (0.174)	−0.235 (0.181)	−0.526 (0.226)^**^	−0.557 (0.268)^**^
**Partner's age**				
18–37	Ref	Ref	Ref	Ref
38–57	0.252 (0.232)	0.370 (0.242)	0.703 (0.308)^**^	0.425 (0.379)
58–77	1.214 (0.608)^*^	1.222 (0.614)	1.302 (0.710)	0.282 (0.766)
>77	−0.189 (0.558)	−0.172 (0.584)	−1.379 (0.554)^**^	−0.192 (0.846)
**Partner's education**				
None	Ref	Ref	Ref	Ref
Primary	0.347 (0.270)	0.550 (0.283)	0.228 (0.385)	−0.271 (0.448)
Secondary	0.628 (0.277)^**^	0.585 (0.284)^*^	0.121 (0.385)	−0.078 (0.465)
Higher	0.820 (0.389)^**^	0.867 (0.404)^*^	−0.029 (0.478)	−0.267 (0.589)
**Partner's occupation**				
Not working	Ref	Ref	Ref	Ref
Working	−0.223 (0.301)	−0.372 (0.306)	0.163 (0.360)	0.408 (0.392)
**Decision on healthcare**				
Self				
Partner	Ref	Ref	Ref	Ref
Both	0..001 (0.354)	0.220 (0.352)	−0.359 (0.437)	0.834 (0.521)
Others	−0.350 (0.356)	−0.121 (0.354)	−0.866 (0.435)	0.464 (0.514)
**Payment for healthcare**				
Self	Ref	Ref	Ref	Ref
Partner	0.314 (0.299)	0.532 (0.302)	0.933 (0.361)^**^	−0.238 (0.515)
Both	0.774 (0.350)^**^	0.802 (0.351)^**^	0.955 (0.415)^**^	−0.179 (0.554)
Others	−0.588 (1.023)	0.290 (1.192)	−0.570 (1.064)	−0.224 (1.218)
**Previous preg. comp**.				
Yes	Ref	Ref	Ref	Ref
No	0.700 (0.198)^***^	0.654 (0.207)	0.640 (0.253)^**^	0.663 (0.300)^**^
No of obs.	1345	1353	1352	1352
Chi2	112.32	96.84	70.59	47.29
Pseudo R2	0.1039	0.0961	0.0976	0.0877

* *p < 0.05*.

** *p < 0.01*.

*** *p < 0.001*.

*Statistical significance at α = 0.05*.

**Table 7:** Self-esteem and utilization of antenatal care, skilled birth care, postnatal care and child vaccination.

**Variables**	**Antenatal care**	**Skilled birth care**	**Postnatal care**	**Child vaccination**
	**Coeff (S.E)**	**Coeff (S.E)**	**Coeff (S.E)**	**Coeff (S.E)**
Self-esteem	−0.224 (0.213)	−0.150 (0.086)	−0.317 (0.104)^***^	−0.861 (0.362)^**^
**Age**				
15–24	Ref	Ref	Ref	Ref
25–34	0.209 (0.268)	0.076 (0.284)	0.134 (0.346)	0.191 (0.413)
35–44	0.350 (0.316)	0.103 (0.331)	−0.436 (0.413)	−0.410 (0.506)
>44	0.722 (0.423)	0.586 (0.451)	0.039 (0.534)	0.484 (0.702)
**Education**				
None	Ref	Ref	Ref	Ref
Primary	0.425 (0.253)	0.413 (0.266)	−0.497 (0.405)	0.416 (0.434)
Secondary	0.924 (0.327)^***^	0.877 (0.342)^**^	−0.455 (0.484)	0.201 (0.541)
Higher	0.798 (0.670)	0.463 (0.648)	−0.774 (0.746)	−0.823 (0.857)
**Marital status**				
Married	Ref	Ref	Ref	Ref
Not married	0.746 (0.187)^***^	0.840 (0.199)^***^	−0.367 (0.233)	−0.984 (0.295)^***^
**Occupation**				
Not working	Ref	Ref	Ref	Ref
Working	−0.252 (0.249)	−0.076 (0.252)	−0.611 (0.335)	0.081 (0.360)
**Literacy**				
Cannot read	Ref	Ref	Ref	Ref
Can read a bit	0.066 (0.207)	0.023 (0.218)	0.589 (0.280)^**^	0.116 (0.341)
Can read well	0.416 (0.347)	0.447 (0.369)	0.275 (0.397)	0.586 (0.537)
**Religion**				
Christianity	Ref	Ref	Ref	Ref
Islam	−0.305 (0.426)	−0.603 (0.411)	0.393 (0.762)	−0.298 (0.774)
Others	−0.336 (0.590)	0.774 (0.816)	−0.110 (0.915)	−0.728 (0.822)
**Parity**				
1	Ref	Ref	Ref	Ref
2	0.051 (0.214)	0.192 (0.230)	0.780 (0.336)^**^	0.163 (0.349)
3	−0.266 (0.580)	−0.105 (0.647)	−1.075 (0.566)	−0.676 (0.793)
**Family type**				
Monogamy	Ref	Ref	Ref	Ref
Polygamy	−0.228 (0.172)	−0.239 (0.180)	−0.514 (0.227)^**^	−0.559 (0.271)^**^
**Partner**'**s age**				
18–37	Ref	Ref	Ref	Ref
38–57	0.221 (0.228)	0.309 (0.239)	0.641 (0.308)^**^	0.467 (0.376)
58–77	1.211 (0.607)^*^	1.188 (0.613)	1.231 (0.711)	0.309 (0.769)
>77	−0.284 (0.540)	−0.270 (0.571)	−1.410 (0.551)^**^	−0.252 (0.828)
**Partner**'**s education**				
None	Ref	Ref	Ref	Ref
Primary	0.346 (0.270)	0.579 (0.284)^**^	0.346 (0.390)	−0.288 (0.456)
Secondary	0.594 (0.277)^**^	0.545 (0.284)	0.137 (0.387)	−0.100 (0.475)
Higher	0.813 (0.392)^**^	0.841 (0.408)^**^	−0.054 (0.483)	−0.278 (0.601)
**Partner**'**s occupation**				
Not working	Ref	Ref	Ref	Ref
Working	−0.308 (0.301)	−0.440 (0.304)	0.140 (0.357)	0.446 (0.393)
**Decision on healthcare**				
Self	Ref	Ref	Ref	Ref
Partner	0.047 (0.344)	0.197 (0.345)	−0.375 (0.433)	0.906 (0.508)
Both others	−0.260 (0.343)	−0.080 (0.345)	−0.786 (0.430)	0.468 (0.500)
**Payment for healthcare**				
Self	Ref	Ref	Ref	Ref
Partner	0.325 (0.294)	0.541 (0.298)	0.908 (0.362)^**^	−0.212 (0.503)
Both	0.845 (0.343)^**^	0.863 (0.347)^**^	1.039 (0.417)^**^	−0.008 (0.543)
Others	−0.728 (0.978)	0.152 (1.163)	−0.618 (1.054)	−0.779 (1.203)
**Previous preg. comp**.				
Yes	Ref	Ref	Ref	Ref
No	0.629 (0.195)^***^	0.578 (0.206)^***^	0.535 (0.257)^**^	0.623 (0.301)^**^
No of observations	1,345	1,353	1,352	1,352
Chi2	94.21	88.73	77.98	49.25
Pseudo R2	0.0872	0.0881	0.1078	0.0913

* *p < 0.05*.

** *p < 0.01*.

*** *p < 0.001*.

*Statistical significance at α = 0.05*.

In Table 8, we showed the marginal effects of locus of control and self-esteem on the probability of utilizing maternal and child healthcare services, computed at the means of the main explanatory variables. We found that utilization of antenatal care, skilled birth care, postnatal care and completion of child vaccination were negatively associated with locus of control and the utilization of antenatal care and skilled birth care showed significant associations. This confirmed our earlier findings and showed that women with external locus of control were 9.5 percent points less likely to utilize antenatal care and 7.0 percent points less likely to utilize skilled birth care. We also found negative associations between antenatal care, skilled birth care, postnatal care and completion of child vaccination and self-esteem. These associations were significant for utilization of antenatal care, skilled birth care and postnatal care. In comparison with women with high self-esteem, women with low self-esteem were 2.7 percent points less likely to utilize antenatal care, 1.6 percent points less likely to utilize skilled birth care and 3.0 percent points less likely to utilize postnatal care.

**Table 8:** Marginal effects of locus of control and self-esteem on the utilization of antenatal care, skilled birth care, postnatal care and child vaccination.

	**Antenatal care**	**Skilled birth care**	**Postnatal care**	**Child vaccination**
	**Coeff (std. err)**	**Coeff (std. err)**	**Coeff (std. err)**	**Coeff (std. err)**
Locus of control	−0.095^***^ (0.021)	−0.070^***^ (0.021)	−0.009 (0.021)	−0.018 (0.016)
Self-esteem	−0.027^**^ (0.021)	−0.016^**^ (0.203)	−0.030^***^ (0.015)	−0.020 (0.012)

** *p < 0.01*.

*** *p < 0.001*.

*Statistical significance at α = 0.05*.

## Discussion

Our study sought to investigate the influence of locus of control and self-esteem on the utilization of maternal and child healthcare services in two rural Local Government Areas in Nigeria. Investigation of the relationship between health-related outcomes and non-cognitive traits such as locus of control and self-esteem is one aspect of improving health behavior that has gained increasing acceptance in recent years, especially in high-income countries (19, 27). Previous studies, which have examined the influence of locus of control and self-esteem on health-related outcomes and lifestyles, have proved their usefulness in the establishment of priorities and allowed intervention programs to focus on potentially modifiable factors in health-seeking behavior.

Our study found significant associations between locus of control and self-esteem on utilization of maternal and child healthcare services in the study population. We found statistically significant associations between locus of control and utilization of antenatal care, skilled birth care, and completion of child vaccination. We found that women with external locus of control were less likely to utilize maternal and child healthcare services when compared with women with internal locus of control. This was consistent with the findings of Kesavayuth et al. in a study on the impact of locus of control in healthcare utilization in Australia, that as locus of control tended toward externality, utilization of healthcare services tended to decline (21). Our study also found statistically significant associations between self-esteem and utilization of postnatal care and completion of child vaccination. We found that women who had low self-esteem were less likely to utilize the various components of maternal and child healthcare services. This finding was, however, not statistically significant in the utilization of antenatal care and skilled birth care.

With the low utilization of maternal and child healthcare services, much still needs to be done to improve maternal and child health outcomes in Nigeria. It is important to give special attention to utilization of healthcare services in underserved regions of the country such as rural communities. Since there was a significant association between non-cognitive traits and utilization of maternal and child healthcare services in our findings, intervention programs should take cognisance of the fact that this dimension of intrinsic barriers exists and channel effort into identifying the level and orientations of non-cognitive traits in the study participants. This could be done through the establishment of counseling clinics, where the psychological needs of women are identified and addressed confidentially, as issues of psychology and mental health are rarely discussed in many lower-middle-income countries because of the fear of stigmatization (58).

The role of professional counseling in the design of interventions aimed at improving maternal and child healthcare outcomes cannot be overemphasized, especially in low resource settings. For instance, Zeligman et al. in a study of the impact of locus of control on trauma survivors in a university in South Eastern USA found that professional counselors were able to consider individuals' perception of control, their ability to take responsibilities for their actions as well as how they perceived problems and obstacles (59). Another aspect that could be useful in the design of health interventions is the fact that locus of control and self-esteem could be impacted by cultural, religious and environmental factors. For women with external locus of control, interventions to improve healthcare utilization could be channeled through people that have been identified in the various communities as having strong influences and acting as “powerful others” to women. These could be partners/husbands, mothers-in-law, and religious, community, or traditional leaders.

Similar to other studies, we found level of education significantly associated with both locus of control and self-esteem and significantly associated with the utilization of antenatal care and skilled birth care. In this regard, governments at all levels could intensify efforts on increasing the proportion of educated women in various communities, regardless of their socioeconomic or sociodemographic status. This is possible through putting initiatives in place to encourage the enrolment of young girls in school and committing more resources to enhancing adult education in order to give uneducated women the opportunity of receiving formal education. In addition, regular health education sessions on the importance of utilizing skilled maternity care during pregnancy and childbirth should be incorporated. Women should be educated on the necessity of early commencement of antenatal checks, knowing the required number of antenatal care visits and on the importance of completing the required vaccinations for their children. There is also need for stakeholders in the Federal and State Ministries of Health to engage in health promotion and awareness campaigns before and during health intervention programmes and to embark on regular and systematic evaluation and review of national programmes initiated to improve maternal and child healthcare utilization in lower-middle-income countries. Such reviews should be done for assessing not only the effectiveness of the programme as a whole but also effectiveness of the various components.

Although our study did not involve experiments based on affecting behavioral change, understanding human behavior is central to enacting effective policies. The results of this study make significant contributions for reforming the demand-side components of health systems and improving maternal and child health outcomes in Nigeria as it provides new insights into the influence of locus of control and self-esteem as predictors of utilization of maternal and child healthcare services. Low utilization of maternal and child healthcare services, especially in rural communities, is one of the most important challenges facing the country in its efforts to reduce the high rate of maternal and child deaths. Clearly, policies and programmes based on the initiation of psychological counseling units for women and provision of need-tailored interventions through good developmental planning and adequate budgetary allocations that target these specific barriers are critical for the country in its bid to improve women's access to skilled maternity care and reduce the number of maternal and child morbidities and mortalities.

The strength of our study lies on being one of the first to contribute to the behavioral economics literature in lower-middle-income countries by examining the influence of non-cognitive traits such as locus of control and self-esteem on utilization of maternal and child healthcare services in a study population of Nigerian women in selected rural communities. Our main results have important implications for creating effective policy actions. Specifically, women with a strong internal locus of control are more likely to utilize skilled maternal and child healthcare services, while those with an external locus of control might need extra incentives to reach similar uptake of healthcare services utilization. While we cannot claim causality from our estimates, locus of control and self-esteem are nevertheless interesting predictors of maternal and child healthcare utilization in low resource settings. We therefore recommend the inclusion of standardized questions on non-cognitive traits into national surveys in lower-middle-income countries to facilitate more research in this field of knowledge.

Our study has some limitations. One of such limitations was that some of the healthcare outcomes were binary. More knowledge of the relationship between maternal and child healthcare utilization and non-cognitive traits would be possible if more information on the outcome variables were available in the dataset. For example, knowing the frequency and timing of antenatal care visits of women in our study population would have allowed for a more precise understanding of the relationship between the outcome variable and non-cognitive traits. In addition, data on locus of control and self-esteem were collected only at the follow-up survey. While it would be better to have collected data on the variables at both baseline and follow-up periods to enable us determine whether there was a change in utilization of maternal and child healthcare services with regards to these traits, our results remain valid due to the assumption of stability in non-cognitive traits as found in previous studies (19, 28). In our study, we aimed to know why women had not utilized skilled maternal and child healthcare services, despite it being available. Hence, the inclusion of standardized questions on women's non-cognitive traits in the follow-up questionnaire. However, taking into account the stability of locus of control over time as found in the study of Cobb-Clark and Schurer (28) and that of Mendolia and Walker (19) on the stability of non-cognitive traits, we might infer that our participants' locus of control and self-esteem remained constant at both data collection points and this justifies our results.

The main objective of our study was to draw awareness to locus of control and self-esteem as significant predictors of maternal and child healthcare services utilization in low resource settings. Future research should build on the findings and recommendations from our study and devote effort to implementing and translating the knowledge from experimental interventions, aimed at changing health behavior into a broader understanding of the political and policy implications of behavioral economics, particularly in lower-middle-income countries.

## Data Availability Statement

The original contributions presented in the study are included in the article/supplementary material, further inquiries can be directed to the corresponding author.

## Ethics Statement

The studies involving human participants were reviewed and approved by National Health Research Ethics Committee (NHREC) of Nigeria—Protocol Number NHREC/01/01/2007–10/04/2017. Community leaders and household heads in the study settings granted the researchers permission to conduct the study. Participation was voluntary, and all study participants signed written informed consent. Rights to privacy and anonymity were respected throughout the study. The patients/participants provided their written informed consent to participate in this study.

## Author Contributions

All authors listed have made a substantial, direct, and intellectual contribution to the work and approved it for publication.

## Funding

This study is supported by the Cooperation for Development PhD Scholarship, Université catholique de Louvain, and the Institute of Health Research and Society (IRSS), Université catholique de Louvain.

## Conflict of Interest

The authors declare that the research was conducted in the absence of any commercial or financial relationships that could be construed as a potential conflict of interest.

## Publisher's Note

All claims expressed in this article are solely those of the authors and do not necessarily represent those of their affiliated organizations, or those of the publisher, the editors and the reviewers. Any product that may be evaluated in this article, or claim that may be made by its manufacturer, is not guaranteed or endorsed by the publisher.

## Footnotes

^1^Ethical approval for the study was obtained from the National Health Research Ethics Committee (NHREC) of Nigeria – protocol number NHREC/01/01/2007–10/04/2017. Community leaders and household heads in the study settings granted the researchers permission to conduct the study. Participation was voluntary, and all study participants signed written informed consent. Rights to privacy and anonymity were respected throughout the study.

^2^Utilization of antenatal care is 86% as compared to the national averages of 67%, skilled birth care is 88% (against 43%), postnatal care is 93% (against 42%) and child vaccination is 95% (against 28%).
